# Enhancing the sensitivity of micro magnetic resonance relaxometry detection of low parasitemia *Plasmodium falciparum* in human blood

**DOI:** 10.1038/s41598-019-38805-2

**Published:** 2019-02-22

**Authors:** Smitha Surendran Thamarath, Aoli Xiong, Po-Han Lin, Peter Rainer Preiser, Jongyoon Han

**Affiliations:** 1 BioSystems & Micromechanics Interdisciplinary Research Group (IRG), Singapore-MIT Alliance for Research and Technology (SMART) Centre, Singapore, Singapore; 2 School of Biological Sciences, Nanyang Technological University, Singapore, Singapore; 3 Department of Electrical Engineering and Computer Science, Massachusetts Institute of Technology, Cambridge, Massachusetts USA; 4 Department of Biological Engineering, Massachusetts Institute of Technology, Cambridge, Massachusetts USA

## Abstract

Upon *Plasmodium falciparum* infection of the red blood cells (RBCs), the parasite replicates and consumes haemoglobin resulting in the release of free heme which is rapidly converted to hemozoin crystallites. The bulk magnetic susceptibility of infected RBCs (iRBCs) is changed due to ferric (Fe^3+^) paramagnetic state in hemozoin crystallites which induce a measurable change in spin-spin relaxation (transverse relaxation) rate in proton nuclear magnetic resonance (NMR) of iRBCs. Earlier, our group reported that this transverse relaxation rate (R_2_) can be measured by an inexpensive, portable 0.5 Tesla bench top magnetic resonance relaxometry (MRR) system with minimum sample preparation and is able to detect very low levels of parasitemia in both blood cultures as well as animal models. However, it was challenging to diagnose malaria in human blood using MRR, mainly due to the inherent variation of R_2_ values of clinical blood samples, caused by many physiological and genotypic differences not related to the parasite infection. To resolve the problem of baseline R_2_ rates, we have developed an improved lysis protocol for removing confounding molecular and cellular background for MRR detection. With this new protocol and by processing larger volume of blood (>1 ml), we are able to reliably detect very low level of parasitemia (representing early stage of infection, ~0.0001%) with a stable baseline and improved sensitivity using the current MRR system.

## Introduction

In spite of the significant progress in the elimination of malaria, it still remains as a major mosquito-borne infectious disease burden in the world, especially in Africa^1,2^. Malaria is caused by *Plasmodium* species parasite like *Plasmodium falciparum*, *P*. *vivax*, *P*. *malaria*, *P*. *ovale*^3^ and *P*. *knowlesi*^4^. Particularly in sub-Saharan Africa, *P*. *falciparum* causes high mortality rates in children under the age of 5^5,6^. Accurate diagnosis of a malaria parasite infection along with rapid treatment significantly reduces the risk of developing severe disease^7^. In addition, ultrasensitive detection of asymptomatic carriers of the parasite is a critical tool for the control and elimination of the parasite from a particular country or region^8,9^.

Currently, diagnosis of malaria exclusively focuses on the parasite blood stage^10^ with Giemsa stained blood smear microscopy being the gold standard for malaria diagnosis^11^. Using thick smear, this approach is able to detect levels as low as 0.001% of parasitemia (50–100 parasites/µl of blood)^12^. The reliability of this approach depends on the quality of the microscopist and can be prone to human error^12,13^. Alternative diagnostic approaches based on protein biomarkers (dipstick), which are easier to use and able to detect 100–1000 parasites per micro liter^2,14^, often suffer from relatively high rate of false positive results^2,15,16^. Polymerase chain reaction (PCR) based detection is the most sensitive malaria diagnosis method, but requires sophisticated laboratory equipment/reagents/skilled labor, making it less suitable for rapid detection in resource poor settings^17,18^.

**Figure 1:**
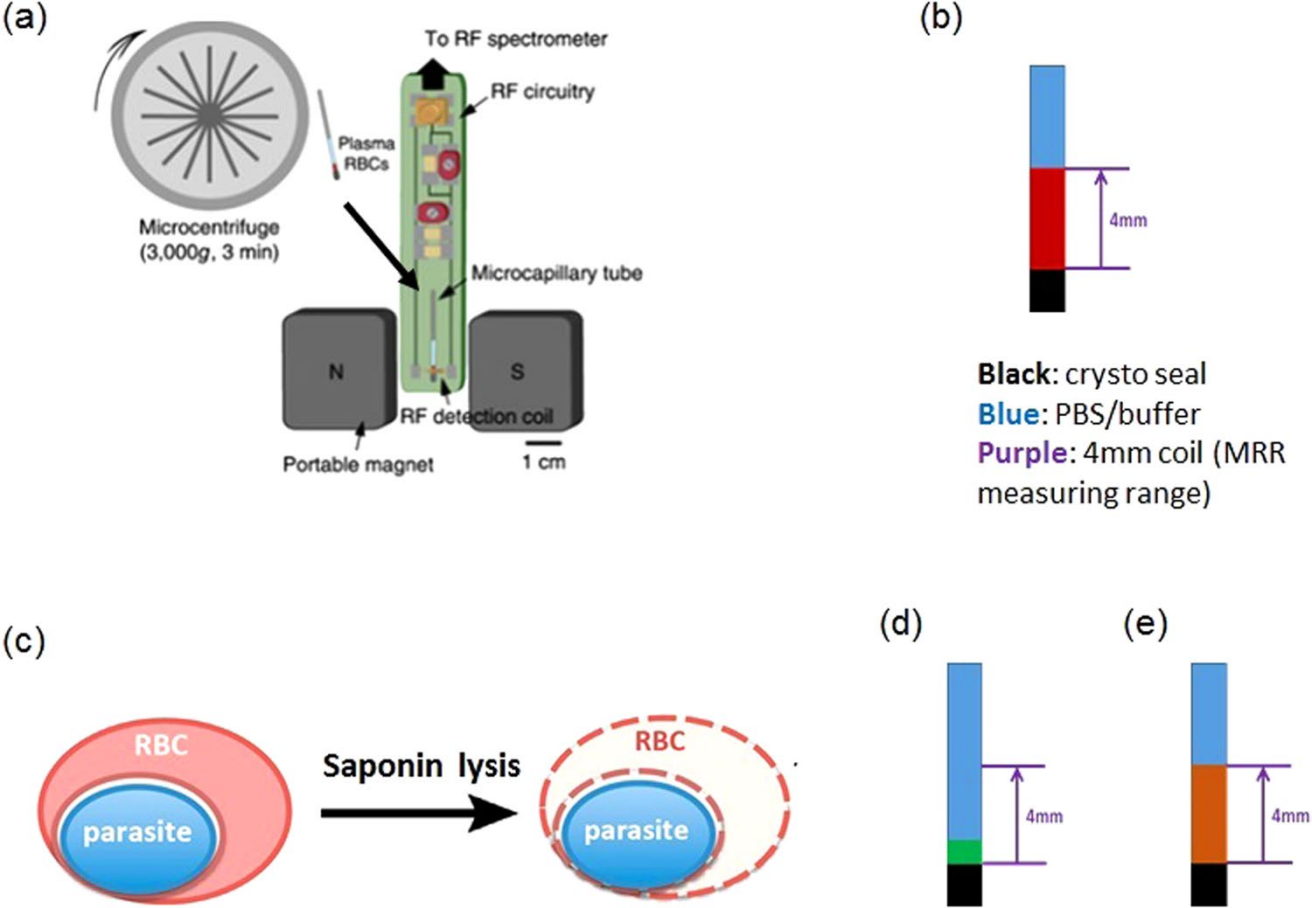
(**a**) Micro MRR system consists of a portable permanent magnet which provides strong magnetic field. A home build radiofrequency (RF) detection probe is connected to RF spectrometer. This probe transmits and receives RF signal from the spectrometer for MRR analysis of the sample inside the RF detection micro coil. The sample, iRBCs/uRBCs pellet was separated from plasma of blood sample to 4 mm detection range of detection coil by using a micro centrifuge (methods)^1^. (**b**) Sample preparation of unlysed uRBCs/iRBCs to micro capillary tube for MRR measurements. Red colour in 4 mm range is the uRBCs/iRBCs pellet separated from PBS (blue colour) by micro centrifuge (methods). (**c**) Saponin lysis of iRBCs (methods)^23^. Sample preparation of lysed uRBCs/iRBCs (**d**) spinning method: spin down all the pellet (green colour) in to a micro capillary tube and (**e**) suspension method: dilute the pellet in 20 µl of PBS and fill the 4 mm detection range (brown colour) of micro capillary tube.

Recently, label free and rapid diagnosis of malaria was possible by using portable benchtop micro magnetic resonance relaxometry (MRR) (Fig. 1a)^1,19^. This is based on the increased hemozoin crystallites being formed in the erythrocyte as the parasites develops from ring, trophozoite to schizont stage (Fig. S1). During blood stage development haemoglobin (Hb) in red blood cells (RBCs) is digested into peptides^20^. The free heme produced is toxic to parasites, and is converted to hemozoin resulting in the diamagnetic Fe^2+^ state of heme changing to Fe^3+^ state in hemozoin^21^. The increased magnetic susceptibility due to paramagnetic hemozoin in iRBCs increases its proton nuclear magnetic relaxation rates (R_2_) relative to the R_2_ rates of healthy RBCs (Fig. S2)^1,19^. The hemozoin crystallites therefore act as a natural, magnetic biomarker for MRR detection of malaria^1,22^. Measuring this increased R_2_ rates in *Plasmodium* species infected blood samples therefore provides a reliable, highly sensitive and fast parasite detection approach allowing detection of early ring stage *P*. *falciparum* infected RBCs with parasitemia as low as 0.0001% (less than 10 parasites/µl). So far, such highly sensitive detection was made possible by comparing the R_2_ rate of iRBCs with that of healthy RBCs from the same source of blood samples^1^, limiting its application to clinical diagnostic settings. The main reason for this is that the R_2_ value of healthy RBCs varies significantly depending on the metabolic conditions of each person, resulting in the possibility of false positive diagnosis due to the presence of different paramagnetic Hb states (*e*.*g*. presence of met-Hb). To overcome this challenge and thereby improve the quality of MRR based diagnosis of malaria in clinical settings, it is necessary to overcome the variations in the baseline R_2_ value that are not directly attributable to parasite infection and growth.

In this work, we report a simple and practical solution to this baseline challenge by the enrichment of *P*. *falciparum* parasites from the iRBCs using saponin lysis, followed by MRR analysis of this enriched parasites for more reliable and sensitive detection. The MRR measurements of saponin lysed healthy RBCs show well-defined and stable baseline R_2_ value, regardless of sample’s metabolic or storage conditions. We have applied two types of sample preparation methods for the measurements of R_2_ using micro MRR that allow rapid and reliable micro MRR detection of iRBCs in as little as 30 minutes. With this new approach, we are able to detect ring stage *P*. *falciparum* iRBCs at 0.001% parasitemia from RBCs equivalent of 100 µl of untreated blood, or 0.0001% in 1 ml of RBCs equivalent of untreated blood. Reliable detection of parasitemia can also be achieved in cryopreserved samples as well as in whole blood containing all the blood cells, with well-defined, universal baseline R_2_ levels. This data shows that micro MRR can be used as an efficient detection and surveillance method for malaria infection in human, in low resource clinical settings.

## Results

### MRR measurement of *P*. *falciparum 3D7* infected red blood cells using different sample preparations

*Plasmodium falciparum* parasites were purified using a Percoll gradient centrifugation to remove uninfected RBCs and obtain a relatively pure schizont stage fraction (approximately 90% schizont stage). The schizont fraction was then diluted with uninfected RBCs (uRBCs) to generate a final parasitemia of 10%. This 10% parasitemia stock solution was subsequently further diluted with uRBCs to create final parasitemia concentrations between 0.0001–10%. Three different approaches to detect a parasite specific signal using the micro MRR (Fig. 1a) were used. For the first approach we measure 100 µl of the iRBCs and corresponding uRBCs (Fig. 1b)^1^. For the other approaches iRBCs (100 µl) were lysed using 0.15% of saponin (Methods) and parasite pellets were collected after repeated washing^23^. Saponin permeablizes the RBC membranes while leaving the malaria parasite membrane intact. By repeated washing after saponin lysis, we can collect a pure parasite pellet. This purified parasite pellet was then processed using two different procedures. For the first procedure (Figs 1d and S5), the parasite pellet was spun down in the micro capillary tube (spinning method) while for the second procedure (Figs 1e and S5), the parasite pellet was diluted in 20 µl PBS and the micro capillary tube was filled with the parasite suspension to the 4 mm detection range (suspension method) (Methods).

### MRR analysis of unlysed and lysed *P*. *falciparum* iRBCs

**Figure 2:**
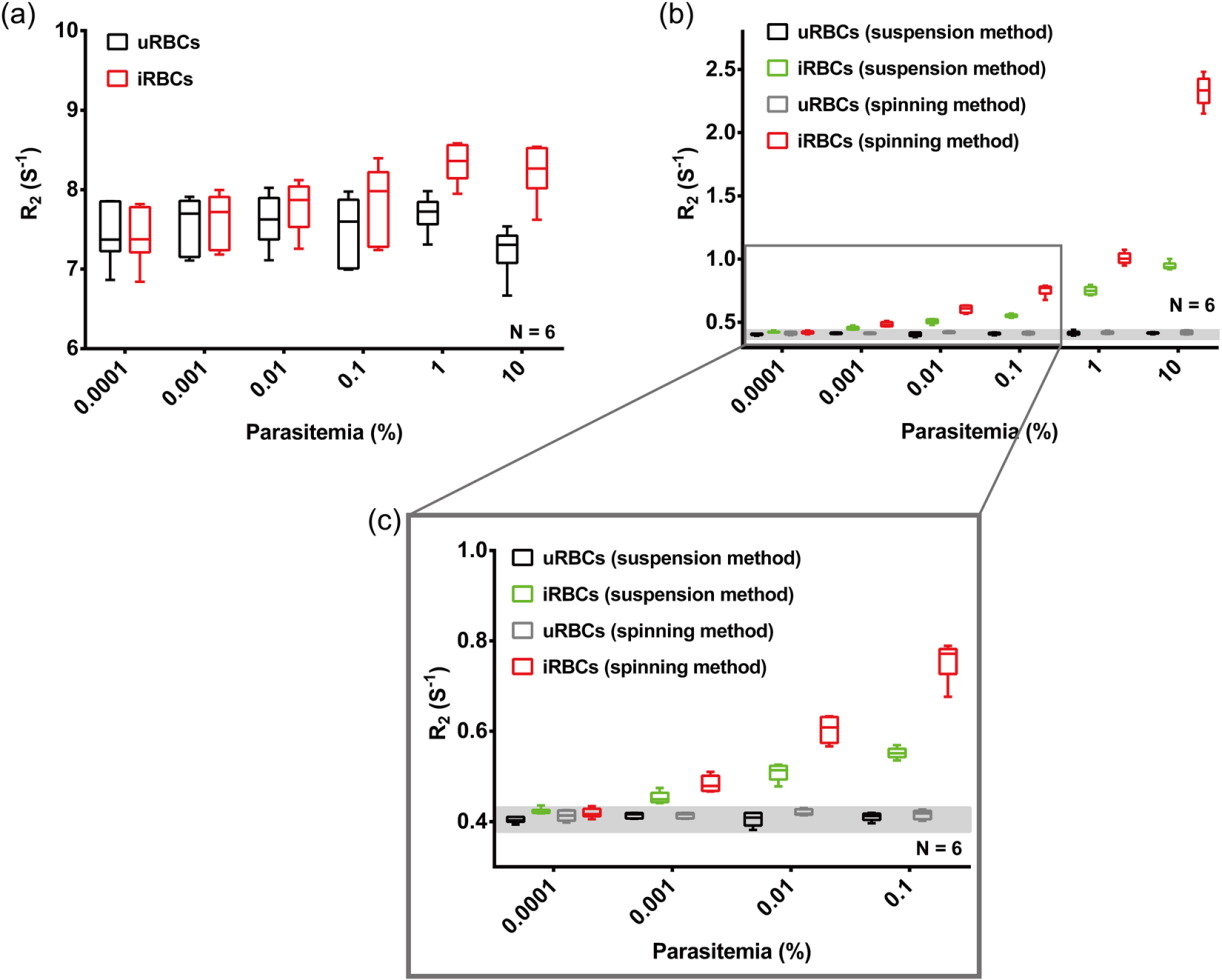
(**a**) R_2_ measurements of unlysed iRBCs spiked to 100 µl of uRBCs to make parasitemia of 10 to 0.0001% (red) and corresponding uRBCs (black) in which blood samples from different healthy blood donors were used. The R_2_ measurements of same set of RBC samples (uRBCs and iRBCs with parasitemia ranging from 10% to 0.0001%) after saponin lysis are shown in (**b**) R_2_ measurements of samples prepared by spinning method (red) and R_2_ measurements of samples prepared by suspension method (green). (**c**) is the enlarged version of (**b**) at low parasitemia region.

To compare the sensitivity of detection of the three different approaches 100 µl of *P*. *falciparum* iRBCs with a parasitemia of 10%, 1%, 0.1%, 0.01%, 0.001% and 0.0001% along with an uRBCs control were analysed to obtain the R_2_ values using the MRR. In addition to account for variations in donor uRBCs that may lead to differences in the measurements obtained parasites were diluted using blood from 6 different donors and the data was shown reflects all the 6 different measurements. As can be seen in Fig. 2a unlysed uRBCs and iRBCs show significant variations in the R_2_ values from donor to donor making it difficult to accurately distinguish iRBCs from uRBCs until parasitemia levels reach between 0.1–1%. The R_2_ value fluctuation of uRBCs (control/baseline values) most likely can be attributed to different metabolic conditions and different uncontrolled Hb states present in the different donors. Saponin lysis and subsequent washing of the samples to obtain purified parasites is a possibility to reduce or remove donor derived fluctuations. As can be seen both the spinning as well as the suspension method (Fig. 2b) show little variation between the R_2_ signal of uRBCs as well as iRBCs from different donors. The uRBCs R_2_ value is stable across multiple measurements and donors and the increase in R_2_ obtained in as little as 0.0001% parasitemia can be clearly distinguished from the uRBCs baseline. Lower R_2_ values of the suspension method compared to spinning method are observed here at each parasitemia level tested. This most likely reflects the fact that in the spinning method (Fig. 1d), the parasite is enriched at the end of the microcappillary tube providing a more concentrated sample at the detection coil (bottom of the microcapillary tube). In the suspension method (Fig. 1e), the enriched parasite is diluted with 20 ul of PBS solution resulting in a less concentrated parasite sample at the detection coil which can measure about 4 ul of the total sample in the microcappillary tube (Fig. S5). Therefore in the suspension protocol, while we would have a lower number of parasites overall they would be more evenly distributed in the detection coil possibly enhancing their detection particularly at low parasitemia. Importantly, there is a clear linear correlation in both methods between the detected R_2_ value and parasitemia which would indicate that it is possible to directly extrapolate parasitemia in a diagnostic sample from a single R_2_ measurement.

### MRR detection of lysed early ring stage *P*. *falciparum* iRBCs

**Figure 3:**
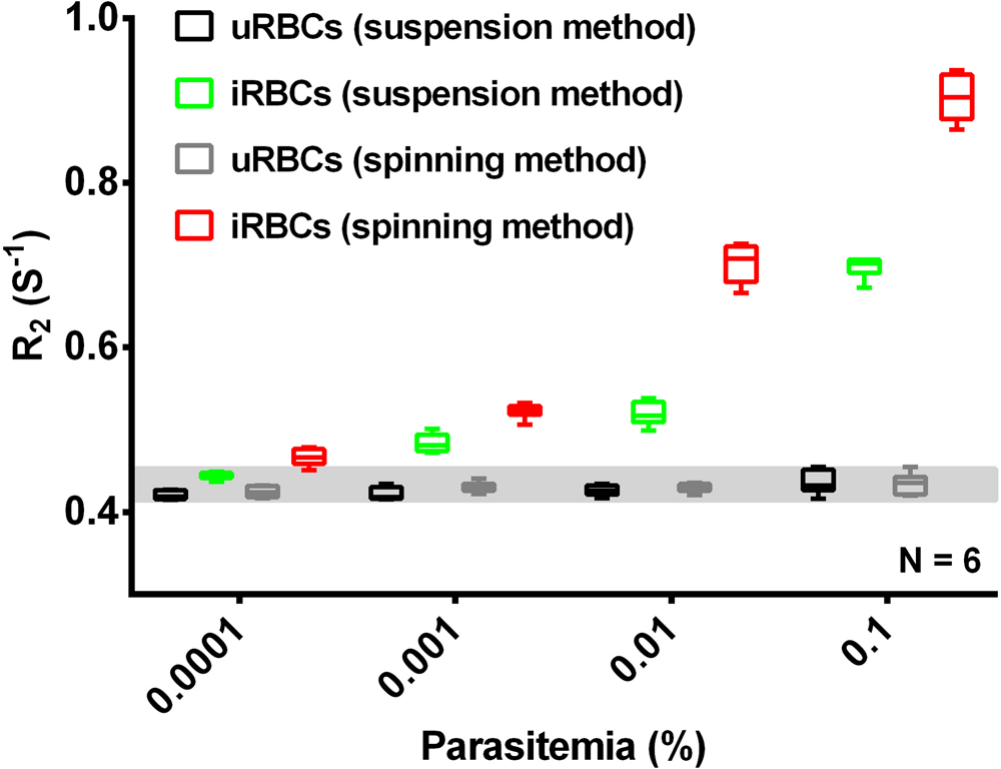
R_2_ measurements of lysed iRBCs/uRBCs using 1 ml of iRBCs (parasitemia ranging from 0.0001% to 0.1%) and uRBCs from different blood sources. R_2_ measurements of lysed iRBCs/uRBCs using different MRR sample preparation by spinning method (red) and suspension method (green).

Under normal circumstances schizonts are not observed in the peripheral circulation of malaria infected patients and it is therefore important to establish whether changes in the R_2_ values detected in ring stage iRBCs are sufficient to allow reliable detection using MRR. As already established previously^1^ ring stage parasites give a lower R_2_ value as compared to schizont stage parasites. Therefore to increase the overall R_2_ signal obtained in a sample we evaluated the sensitivity of our MRR detection when increasing the overall sample volume analysed to 1 ml. For this we prepared synchronized ring stage parasites (Fig. S3a) and diluted them with uRBCs to make 1% parasitemia. This 1% parasitemia ring stage iRBCs was further diluted with uRBCs to make parasitemia of 0.1%, 0.01%, 0.001% and 0.0001%. These samples were then analysed using both the spinning and suspension methods (Fig. 3). As already seen before the uRBCs samples gave a consistent R_2_ reading irrespective of the donor blood used. Importantly, both methods showed a significant difference in the R_2_ value at parasitemia levels as low as 0.0001%. The low R_2_ value in suspension method compared to that of spinning method is explained by the reduced number of parasites in the detection range of microcappillary coil (Fig. S5). The data would indicate that by increasing the amount of iRBCs to 1 ml for saponin lysis, we can detect ring stage parasites observed in peripheral blood circulations to levels as low as 0.0001%.

### Comparison of early ring stage and late schizont stage *P*. *falciparum* iRBCs

**Figure 4:**
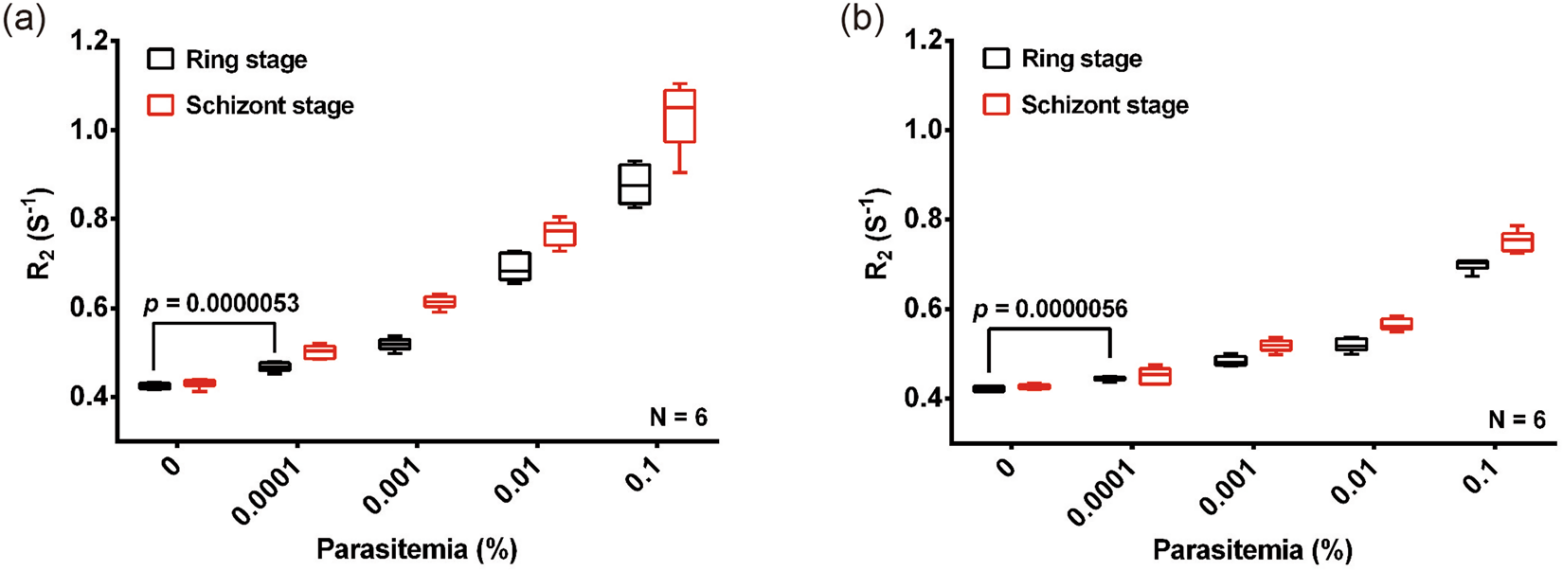
MRR measurements of *P*. *falciparum* iRBCs schizont stage (red) and ring stage (black) spiked to 1 ml of uRBCs. R_2_ values of these iRBCs and uRBCs after saponin lysis and using two types of sample preparation (**a**) by spinning method and (**b**) by suspension method.

To compare the differences in signal obtained from 1 ml of synchronized rings or schizonts we prepared stock solutions with a parasitemia of 0.1%, 0.01%, 0.001%, and 0.0001%. A side by side comparison of both the spinning and the suspension (Fig. 4) methods showed a consistent increase in the R_2_ value with increasing parasitemia. The measured R_2_ value was consistently higher in the schizont sample consistent with a greater content of magnetic crystals of hemozoin in the schizont stage. Moreover, there is again little variation in the R_2_ value observed in all samples irrespective of the donor blood used to prepare the parasite dilutions. There is a significant difference (P value = 0.000005) in the R_2_ value between uRBCs and iRBCs even at the lowest parasitemia levels of 0.0001% used here indicating that the MRR assay coupled to either lysis protocols has the sensitivity needed for very early diagnosis of even asymptomatic parasite carriers.

### Detection of *P*. *falciparum 3D7* infection from frozen lysed iRBCs by MRR

**Figure 5:**
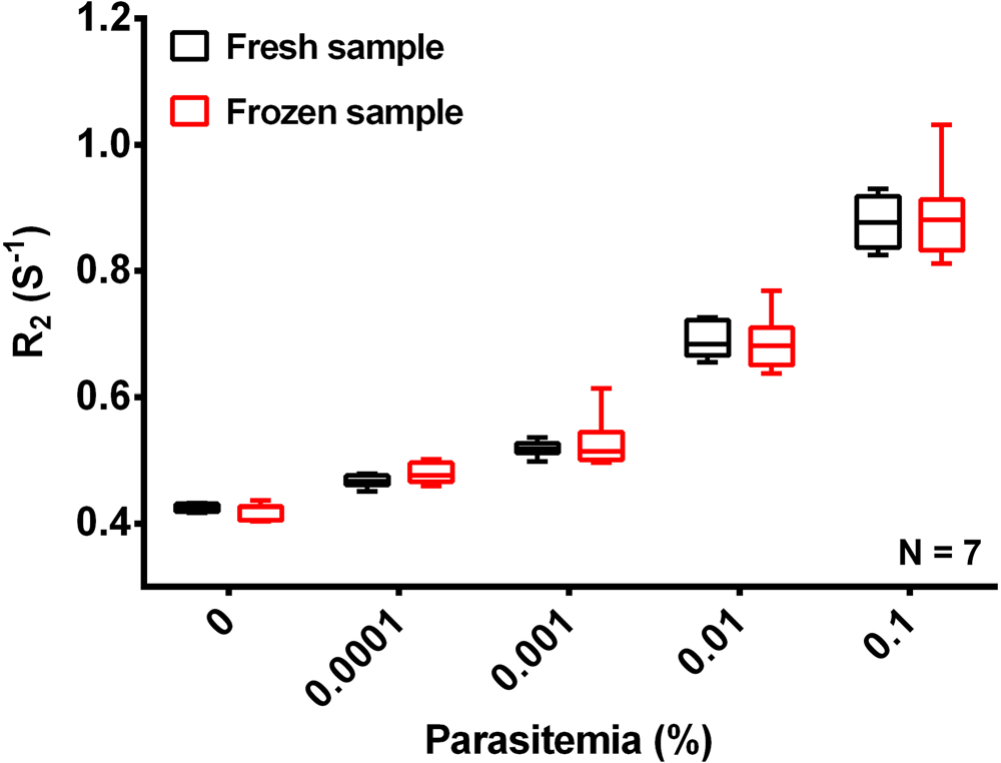
MRR detection of frozen samples of lysed *P*. *falciparum 3D7* ring stage iRBCs. R_2_ values of lysed *P*. *falciparum* ring stage parasites (black). The R_2_ values of cryogenically stored (−140 °C at liquid nitrogen) corresponding set of lysed iRBCs/uRBCs pellets for two weeks (red). All samples were spun down to micro capillary tube (spinning method).

To establish whether long term storage of frozen blood samples impacted on the R_2_ value observed we prepared washed ring stage parasites with parasitemia of 0.1%, 0.01%. 0.001% and 0.0001% as well as uRBCs as above. The pellets were collected after repeated washings and stored in −140 °C at liquid nitrogen for two weeks. These cryogenically stored lysed samples were analysed by MRR and compared to freshly prepared ring stage parasite samples of similar parasitemias (Fig. 5). It is very clearly demonstrated in Figs 2 and 3 that the early stage (0.0001% parasitemia) diagnosis of *P*. *falciparum* is possible by both spinning and suspension methods and both methods shows similar trend in R_2_ value variation with increase in parasitemia. Either spinning method or suspension method is a reasonable choice, we choose spinning method for further MRR measurements. The R_2_ measurements of both freshly prepared samples as well as the frozen samples were comparable to each other and suggests that the R_2_ values are not impacted by long term cryopreservation.

### MRR detection of *P*. *falciparum* iRBCs spiked to fresh blood

**Figure 6:**
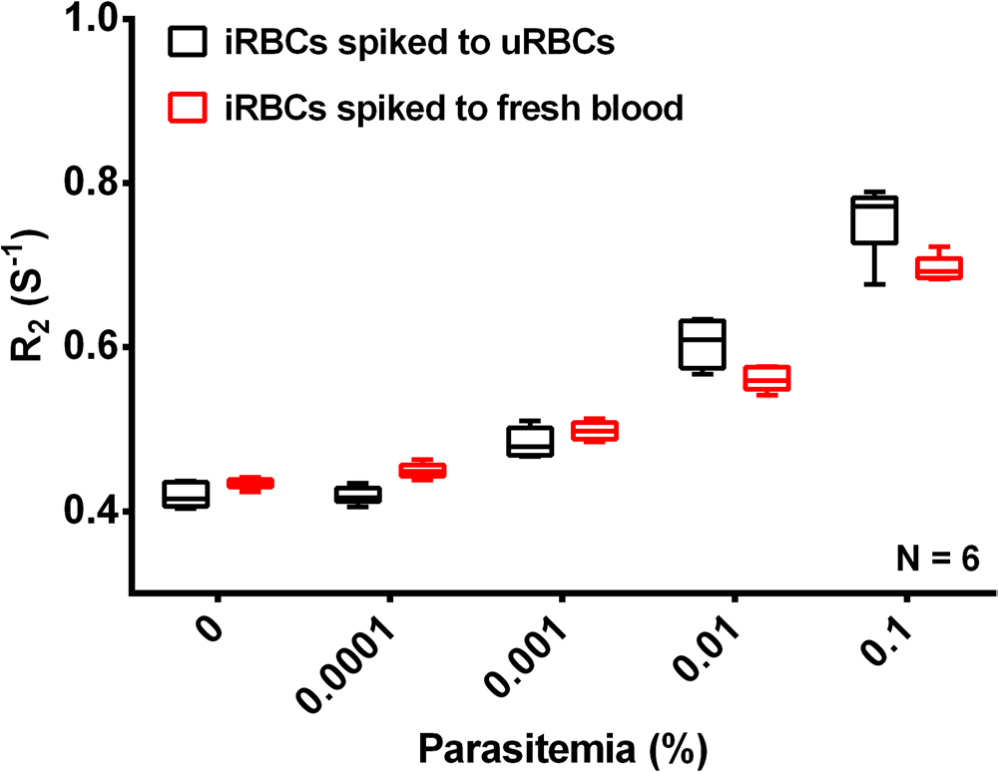
MRR measurements of *P*. *falciparum* spiked fresh blood samples. R_2_ values of lysed iRBCs which is *P*. *falciparum* ring stage parasites spiked to 100 µl of uRBCs (black). R_2_ values of lysed infected fresh blood which is *P*. *falciparum* ring stage parasites spiked to 200 µl of freshly drawn blood samples (red). All samples were spun down to micro capillary tube (spinning method).

In clinical settings a parasite diagnostic test would have to be able to work reliably with whole blood obtained directly from the patient without further purification. To mimic these clinical conditions, we diluted the ring stage *P*. *falciparum* 3D7 iRBCs with freshly drawn blood instead of washed and concentrated uRBCs to parasitemias of 0.1%, 0.01%, 0.001% and 0.0001%. These infected blood samples as well as uninfected fresh blood (0% parasitemia) were lysed by saponin and the pellet was collected after washing. The R_2_ values of the different samples were determined by MRR indicating that there is no significant difference in limit of detection of malaria for the samples diluted with purified uRBCs (100 µl) or whole fresh blood (200 µl) (Fig. 6). As stated earlier, the limit of detection could be increased to 0.0001% by using 1 ml infected whole blood sample. These results indicate that highly sensitive detection of parasites using the MRR diagnosis approach is feasibly with freshly drawn blood samples normally obtained in clinical settings.

## Discussion

The reliable diagnosis of malaria at the earliest stage of infection is an essential tool for the reliable treatment of infected individuals. The detection of asymptomatic carriers with very low levels of parasitemia is critical for the global effort to eliminate malaria. In addition, with the recent rise in drug-resistant parasites in certain parts of the world^2^, it is also important to monitor the efficacy of drug treatment as a function of time, by measuring the active parasite burden directly, repeatedly, rapidly, and preferably at a low cost. Current malaria diagnostic options, including Giemsa microscopy, protein marker based malaria rapid diagnostic tests (RDTs), as well as PCR based detection, fall short of meeting all of the above diagnostic requirements for the future global malaria eradication efforts^10^. MRR based detection of malaria parasite described in this paper provides a potential viable tool to support the malaria eradication effort as it is rapid, sensitive, and cost effective while having the additional benefit of being suitable for automation. In addition, the R_2_ value obtained by MRR can be regarded as an alternative quantitative metric replacing parasitemia, which can be used for time-dependent monitoring of the live, active parasite burden for managing drug-resistant malaria^1,19^. Yet, in order to realize the full potential of MRR, it is necessary to overcome host specific variations in blood chemistry, variations in blood collection and storage in field clinics and other factors that could directly impact on the resulting R_2_ measurements independent of the parasite burden. Here we report a simple strategy that allows us to eliminate most of the non-parasite derived factors that would contribute to variations of the R_2_ measurements. This approach allowed the reliable detection of live parasites independently of host factors and is suitable to evaluate even frozen samples.

The saponin treatment utilized in our protocol permeablizes the membranes of iRBCs and uRBCs while leaving the parasite membrane intact^23^. Combining this with a rigorous washing step allowed the removal of most of the host cell contents thereby removing potential confounding factors like cell-free Hb, RBC ghosts, white blood cells, etc., leaving only the parasite pellet behind (Fig. S4). Based on this concept two slightly different approaches were used to prepare the parasite pellet for MRR analysis: (1) spinning method: Spin down the pellet into the bottom of a micro capillary tube. (2) suspension method: Dilute the pellet in 20 µl of PBS and filled in the 4 mm detection range of a micro capillary tube (Methods and Fig. S5). Both approaches greatly improve the ability of MRR to detect the parasite and to remove confounding factors with the suspension method providing higher detection sensitivity and a lower limit of detection (LOD), possibly as the very tiny parasite pellets (Fig. 2C), spun down at the bottom of the micro capillary (at lower parasitemia), was not properly aligned to be measured by the micro coil.

This parasite pellet enrichment approach developed here has a profound impact on both the sensitivity as well as the reliability of the MRR based diagnostic approach. Firstly, the method now shows a uniform baseline R_2_ index in blood samples of different donors (Fig. 2) thereby removing the challenge of the baseline R_2_ value. Secondly, by increasing the sample volume tested to 1 ml of infected blood the MRR allows reliable detection of parasitemia of 0.0001%, which would represent the early asymptomatic stage of the infection. Importantly, high sensitivity of detection is obtained even with ring stage parasite confirming the suitability of the MRR approach to detect parasites in blood obtained from the peripheral circulation of infected patients. While recent progress on next-generation malaria detection based on PCR amplification^24^ is advancing our ability to monitor malaria burden in a given population, we believe that MRR approach would complement any other high-sensitivity diagnostics methods of the future, simply because the R_2_ are not based on molecular signature of parasites, but on the metabolic waste production resulting from parasite growth.

The relatively large volume (100 µl/1 ml) of blood needed for the MRR diagnostics of malaria is a potential drawback as both Giemsa staining and RDT require a much smaller volume of blood. However, this is offset by the significantly higher sensitivity and range of detection, with Giemsa staining becoming unreliable when the parasitemia number is below 0.1%^25^. If the MRR diagnostic method can provide repeatable, rapid, and reliable measurement of parasite burden 2–3 orders of lower magnitude, then the need for an increased blood volume would be justified especially as the required volume of 1 ml is well within the acceptable range of for many blood based diagnostic tests. The ease of sample preparation and measurement of R_2_ values using MRR also offer a significant benefit as well as the low cost (approximately 10 cents per assay) making it appropriate for malaria endemic countries. However, the current MRR system is still expensive and its electronics and polarizing magnets are the major cost drivers of this MRR instrument. However, the recent developments of on-chip NMR systems^26^ using inexpensive magnets and electronics will significantly reduce the cost of the detection equipment needed to below $500.

In conclusion, the results obtained in this study significantly strengthen the applicability of MRR as an efficient diagnostic method for malaria in clinical settings. Using the current enrichment method, sample preparation and use of MRR for diagnosis, more effective surveillance of this disease in malaria endemic regions will be possible.

## Methods

### *In vitro* culture of *P. falciparum* 3D7

Blood stage *P*. *falciparum* 3D7 parasites (MR4, USA) were grown using human RBCs in RPMI 1640 (Life technologies) supplemented with 0.25% AlbuMAX II (Invitrogen, USA), 2.3 g/L sodium bicarbonate (Sigma-Aldrich, USA), and 0.1 mM of hypoxanthine (Sigma-Aldrich, St. Louis, USA) 10 mg/L of gentamicin (Invitrogen, USA). The culture was maintained at 2.5% haematocrit at 37 °C, 5% CO_2_, 3% O_2_ and 92% N_2_.

Synchronized early ring-stage parasites were obtained using 5% D-sorbitol^27^, while schizont stage parasites were purified using 68% Percoll centrifugation as described^28,29^. Parasitemia was determined by standard Giemsa stain microscopy. For preparation of different parasitemia samples, iRBCs were spiked into uRBCs to obtain different final parasitemia. Fridge stored blood and freshly drawn blood were used based on different experiment design.

### Saponin lysis protocol and sample preparation for MRR

For enrichment of parasites from blood, 0.15% saponin was added to the RBCs in the volume ratio of 3 to 1^23^. The sample was kept on ice for 15 minutes and vortexed every few minutes. The sample was then washed with PBS thrice and centrifuged at 5000 g for 5 min. After washing, sample pellet was collected and filled into micro capillary tube for MRR experiments. For experiments with cryogenically stored samples, RBCs were lysed with 0.15% saponin and washed as described above. The processed sample was then stored in −140 °C at liquid nitrogen for up to two weeks. Before MRR experiments, all the samples were thawed and brought back to room temperature.

For preparation of unlysed samples, the spiked iRBCs/uRBCs mixture was collected and adjusted to 10% haematocrit. The cell suspension was then filled into the micro capillary tube (22-260-950, Fisherbrand, Waltham, MA, USA) for 4 cm length and sealed with an inert seal (Critoseal, Krackeler Scientific Inc., Albany, NY, USA). The micro capillary tube was centrifuged at 3000 g, 3 min in a micro centrifuge (Sorvall Legend Micro 21, Waltham, MA, USA) to make a 4 mm band of compact iRBCs/uRBCs which is in the detection range of the micro coil (Fig. 1a,b).

For the preparation of saponin lysed samples, lysed pellet was diluted in 20 µl of PBS and filled in to micro capillary tube. For spinning method sample preparation, the micro capillary tube was sealed and centrifuged at 5000 g for 5 min to concentrate the saponin lysed pellet in to the 4 mm detection range of the micro coil (Fig. 1d). For suspension method sample preparation the collected pellet was diluted in 20 µl of PBS and vortexed to make a uniform solution. The solution was then filled in to the 4 mm range of the micro capillary tube (Fig. 1e). MRR measurements of this sample was done immediately without spinning the micro cappillary tube. This suspension method detection is repeated 3 times from the same solution and the average R_2_ value was calculated.

### MRR measurements and detection

MRR consists of a portable permanent magnet (Metrolab Instruments, Plan-les-Ouates, Switzerland) with *B*_0_ = 0.5 T and a bench-top type NMR console (Kea Magritek, Wellington, New Zealand). ^1^H MRR measurements were performed at the resonance frequency of 21.65 MHz inside the magnet^1^. A single resonance proton MRR probe with detection micro coil of 900-μm inner diameter was used for accommodating the MRR samples in to the micro capillary tubes (o.d.: 1,500 μm, i.d.: 950 μm) (22-260-950, Fisherbrand, Waltham, MA, USA). In MRR probe, the electronic parts and coil were mounted on the single printed circuit board (Fig. 1a)^1^. All the experiments were performed at 26.3 °C inside the magnet which is maintained by a temperature controller (RS component, UK).

Proton transverse relaxation rates R_2_ were measured by standard Carr-Purcell-Meiboom-Gill (CPMG) pulse programme (Fig. S6)^30,31^. We maintained the transmitter power output at 12.5 mW for a single 90° pulse of pulse length 16 μs for all the R_2_ measurements. The CPMG train of pulses with inter echo time of 60 μs with 4000 echoes were used for the experiments stated in Fig. 2a. A recycle delay of 2 s, which is sufficient to allow all the spins to return to thermal equilibrium, was used. 64 scans were performed for all experiments for signal averaging. For experiments using saponin lysed samples, different recycle delay ranging from 4 s to 15 s were used based on different parasitemia.

### Ethics statement

All experiments were performed in accordance with relevant guidelines and regulations. The use of human blood was approved by the domain-specific review board of Nanyang Technological University (IRB number: IRB-2017-03-020). Blood component collection service was provided by Blood Transfusion Service and Blood Donation Centre of National University Hospital. All individuals gave informed consent.

## Supplementary information

Enhancing the sensitivity of micro magnetic resonance relaxometry detection of low parasitemia *Plasmodium falciparum* in human blood

## Data Availability

The datasets generated during and/or analyzed during the current study are available from the corresponding author on reasonable request.
