# Supplementary material for: Enhancing the sensitivity of micro magnetic resonance relaxometry detection of low parasitemia Plasmodium falciparum in human blood
Source: Sci Rep. 2019 Feb 22;9:2555. doi: 10.1038/s41598-019-38805-2 (PMC6385492; doi:10.1038/s41598-019-38805-2)
Supplement: Supplementary file 1 — Enhancing the sensitivity of micro magnetic resonance relaxometry detection of low parasitemia Plasmodium falciparum in human blood [file 41598_2019_38805_MOESM1_ESM.pdf]

Supplementary Information for

Enhancing the sensitivity of micro magnetic resonance relaxometry detection of low parasitemia *Plasmodium falciparum* in human blood

Smitha Thamarath Surendran,[1] Aoli Xiong,[1,2] Po-Han Lin,[1,3] Peter Preiser,[2] Jongyoon Han,[1,3,4]*

[1]BioSystems & Micromechanics Interdisciplinary Research Group (IRG), Singapore-MIT Alliance for Research and Technology (SMART) Centre, Singapore.

[2]School of Biological Sciences, Nanyang Technological University, Singapore.

[3]Department of Electrical Engineering and Computer Science, Massachusetts Institute of Technology, Cambridge, Massachusetts, USA.

[4]Department of Biological Engineering, Massachusetts Institute of Technology, Cambridge, Massachusetts, USA.

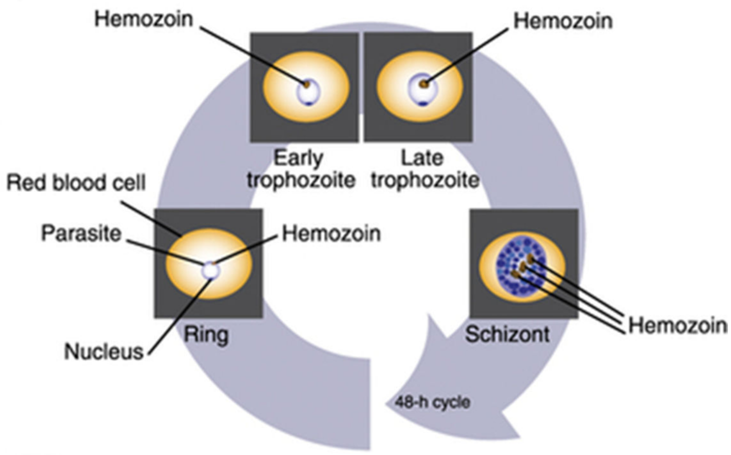

Figure S1: 48 hour intraerythrocyte cycle of *P. falciparum* 3D7[1]: When merozoites infects nRBCs, the haemoglobin in nRBCs are converted to hemozoin and this pigments are deposited in the vacuole of erythrocyte to form early ring stage. It matured to early trophozoite, late trophozoite and schizont stage in 48 hour.

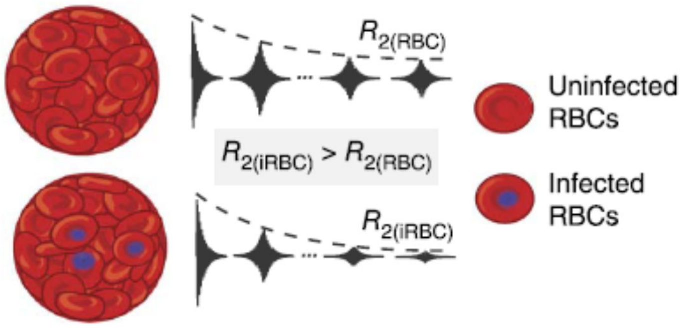

Figure S2: Proton transverse relaxation rate (R_2) of infected RBCs and uninfected RBCs (uRBCs).[1] Due to the presence of paramagnetic hemozoin in iRBCs, their proton relaxes faster than the protons of nRBCs. This decrease in R_2 value in iRBCs is directly proportional to the parasitemia increase in iRBCs.

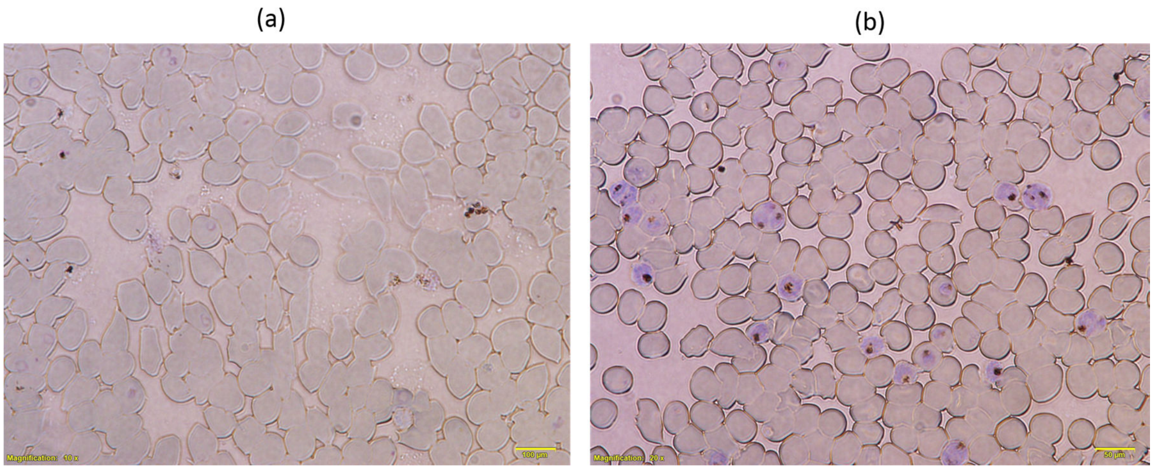

Figure S3: The ring stage of *P. falciparum 3D7* iRBCs after sorbitol treatment (a) and the schizont stage of *P. falciparum* 3D7 iRBCs (b).

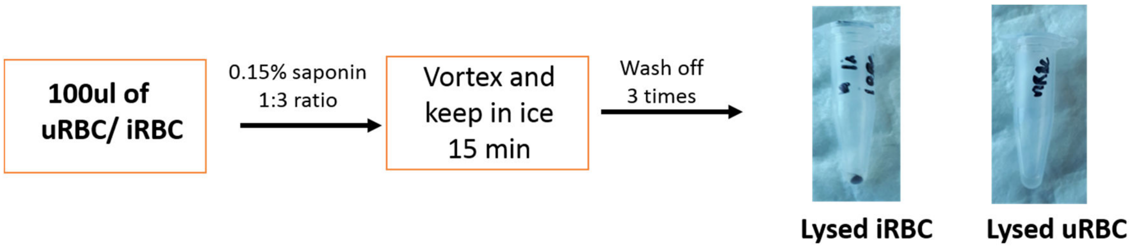

Figure S4: saponin lysis protocol of uRBCs/iRBCs

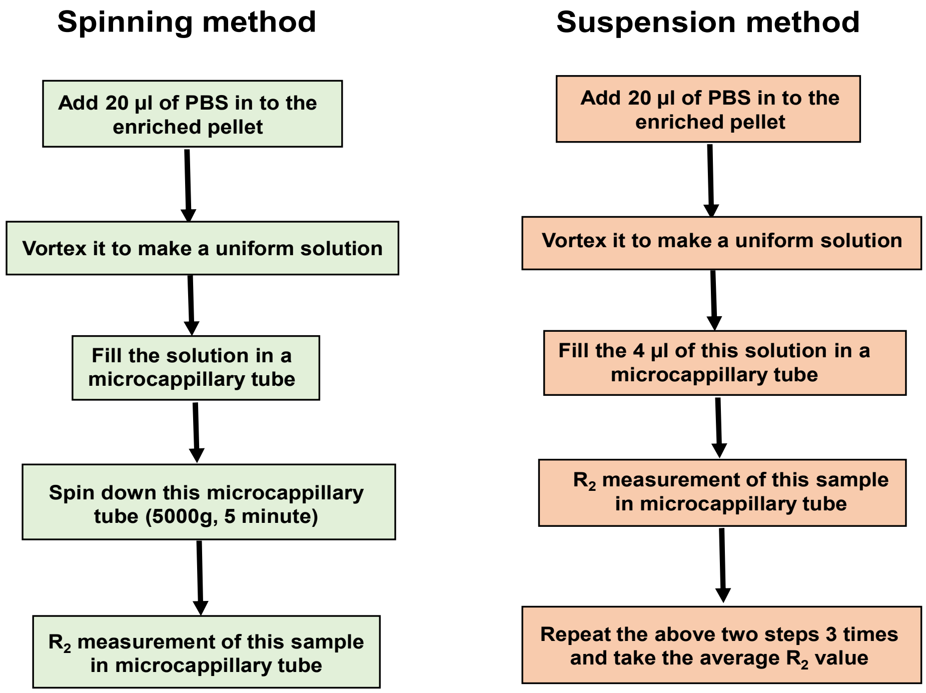

Figure S5: Protocol for MRR sample preparation spinning method and suspension method

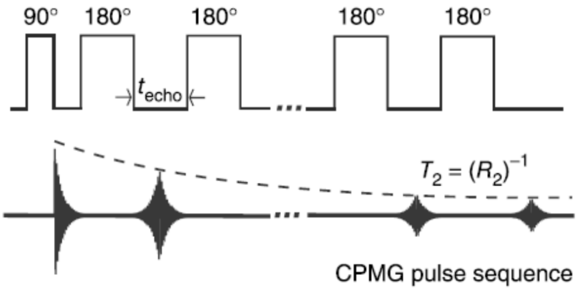

Figure S6: The CPMG (Carr-Purcell-Meiboom-Gill) pulse sequence[2,3] for measuring the R_2 rates of iRBCs/nRBCs which works efficiently in inhomogeneous magnetic fields produced by permanent magnet of MRR.[1] A train of radiofrequency pulses are applied to the proton nuclei at the resonance frequency of 21.65 MHz with the inter echo time interval t_{echo} and it is repeated for thousands of echoes until relaxes over time. This decayed height of echoes over time is called transverse relaxation time T_2 which is inversely proportional to R_2.

References

1 Peng, W. K. *et al.* Micromagnetic resonance relaxometry for rapid label-free malaria diagnosis. *Nat Med* **20**, 1069-1073, doi:10.1038/nm.3622 (2014).

2 Carr, H. Y. & Purcell, E. M. Effects of Diffusion on Free Precession in Nuclear Magnetic Resonance Experiments. *Phys. Rev* **94**, 630–638 (1954).

3 Meiboom, S. & Gill, D. Modified Spin-Echo Method for Measuring Nuclear Relaxation Times. *Rev. Sci. Instrum* **29**, 688–691 (1958).
